# Person-related work and the risk of type 2 diabetes: a Swedish register-based cohort study

**DOI:** 10.1136/oemed-2025-110088

**Published:** 2025-06-24

**Authors:** Kuan-Yu Pan, Alicia Nevriana, Melody Almroth, Daniel Falkstedt

**Affiliations:** 1 Insitute of Environmental Medicine, Karolinska Institutet, Stockholm, Sweden

**Keywords:** Epidemiology, Occupational Health, Diabetes mellitus, Longitudinal studies, Occupational Groups

## Abstract

**Objectives:**

Person-related work requires workers to interact with individuals not employed at the workplace, such as clients and patients, and can cause emotional labour and conflict. These stressors may increase workers’ risk of type 2 diabetes, but their impact may differ depending on the level of support received from colleagues. We aimed to examine the association between person-related work and the risk of type 2 diabetes, and the effect modification of social support at work.

**Methods:**

The study population consisted of around three million people without type 2 diabetes in Sweden in 2005, who were employed and aged 30–60 years. Three dimensions of person-related work—general contact with people, emotional demands and confrontation—and social support were respectively assessed using job exposure matrices. Patients with type 2 diabetes in 2006–20 were determined based on drug, patient and death registers. Multivariable Cox regression models were used.

**Results:**

High exposures to emotional demands and confrontation were respectively associated with 20% and 15% increased risks of type 2 diabetes in men and 24% and 20% in women. In both men and women, there was statistically significant effect modification by social support—the associations between emotional demands and confrontation and type 2 diabetes were higher among those with low social support than those with high social support.

**Conclusions:**

In both men and women, dimensions of person-related work, including emotional demands and confrontation, are associated with an increased risk of type 2 diabetes, and low social support at work seems to amplify the magnitude of these associations.

**WHAT IS ALREADY KNOWN ON THIS TOPIC**

- Adverse psychosocial working conditions are associated with a higher risk of type 2 diabetes. However, the potential effect of exposures within person-related work on type 2 diabetes has not been studied.

**WHAT THIS STUDY ADDS**

- Dimensions of person-related work, including emotional demands and confrontation, are associated with an increased risk of type 2 diabetes, and low social support at work amplifies the magnitude of these associations.

**HOW THIS STUDY MIGHT AFFECT RESEARCH, PRACTICE OR POLICY**

- Our findings suggest that preventive strategies aiming to reduce the risk of type 2 diabetes may be developed for workers in person-related work.

## Introduction

 The prevalence of type 2 diabetes has been increasing worldwide.^1^ Type 2 diabetes can lead to various complications, and thus identifying modifiable risk factors for type 2 diabetes is an important approach to prevent further health deterioration from the condition.^2^ Existing evidence has linked adverse psychosocial working conditions, such as job strain,^3 4^ job insecurity,^5^ workplace violence and bullying,^6^ and effort–reward imbalance,^7^ to a 10–60% higher risk of developing type 2 diabetes. However, the potential effect of exposures within person-related work on type 2 diabetes has not been studied.

Person-related work refers to occupations that require face-to-face or voice-to-voice interaction with individuals who are not employed at the workplace (ie, a third party), such as patients, customers, clients, passengers or students.^8^

Various aspects of person-related work may cause stress, which can subsequently increase the risk of type 2 diabetes.^9^ First, frequent *contact with people* who are not employed at the workplace requires workers to constantly regulate their emotions to align with organisational expectations. This emotional labour can be especially taxing when there is a discrepancy between displayed emotions and those genuinely felt.^8^ Second, high *emotional demands* are common in certain person-related work, such as healthcare, social services and education (ie, human service occupations).^10^ These roles require empathy and emotional engagement with third parties who are often in difficult or distressing situations.^11^ Third, workers in these occupations frequently address needs and problems of customers, which can sometimes result in *confrontation*.

Besides the pathophysiological changes related to stress response, stressors in person-related work may also lead to behavioural changes, such as overeating, physical inactivity^12^ or excessive alcohol consumption.^13^ These behaviours can also contribute to the risk of type 2 diabetes in person-related work.

The quality of emotional and instrumental social interaction is an important characteristic of workplaces,^14^ and stress levels in person-related work may vary depending on the amount of social support provided by supervisors and colleagues.^15^ While low emotional support at work has been associated with a higher risk of type 2 diabetes,^16^ favourable workplace psychosocial resources, including leadership and support from colleagues, have been associated with a lower risk of type 2 diabetes.^17^ Therefore, it is plausible that social support at work plays a moderating role for the risk of type 2 diabetes in person-related work.

This study aimed, for the first time, to investigate the prospective relationship between three dimensions of person-related work—general contact with people, emotional demands and confrontation—and the risk of type 2 diabetes. Furthermore, it examined whether social support at work moderates this relationship. We hypothesised that higher exposures to these three dimensions are associated with a higher risk of type 2 diabetes and that the increased risk is more prominent among workers who receive lower social support at work.

## Research design and methods

### Study population

The study population was extracted from the Swedish Work, Illness, and labour-market Participation (SWIP) cohort which consists of around 5.4 million individuals aged between 16 and 65 years and registered in Sweden during the baseline year of 2005. Data were retrieved from several Swedish administrative and medical registers, including the total population register,^18^ the longitudinal integrated database for health insurance and labour market studies (LISA) register,^19^ the national patient register,^20^ the prescribed drug register,^21^ the cause of death register, as well as information from earlier population censuses. Linkages between registers were made by Statistics Sweden using unique personal identification numbers.

The present study included individuals who were aged 30–60 years, had information on job held in 2005, and had no history of any type of diagnosed diabetes (International Classification of Diseases (ICD)-8 code 250, ICD-9 code 250 and ICD-10 codes E10-14) or a prescription of antidiabetic drugs (ATC code A10) in or before 2005. This resulted in a final population of 2 950 186 individuals, of which 50.9% were women.

### Exposure

Yearly occupational information is available in the LISA register administered by Statistics Sweden from 2005 onwards, documented based on the Swedish ISCO-88 four-digit classification of occupations. We extracted individuals’ occupational codes in 2005.

We assessed three dimensions of person-related work using a job exposure matrix (JEM) based on the Swedish Work Environment Surveys (1997–2013). The questions and answer options available in these surveys are shown in table 1. For around 350 occupations, we calculated the proportion of responses to a certain level of exposure, separately for men and women. For the general contact with people dimension, similar to a previous study,^22^ we calculated the proportion of responses to ‘roughly ¾ of the time’ or ‘almost all the time’. For emotional demands and confrontation, we calculated the proportion of responses to ‘a few days per week’ or ‘every day’.

**Table 1:** Questions and answer options in the Swedish Work Environment Surveys (1997–2013) by dimensions of person-related work

Dimension	Question	Answer option
General contact with people	Do you have anything to do with people at work who are not employed at the workplace (eg, patients, customers, clients, passengers, students, etc)?	Not at all, roughly 1/10 of the time, roughly ¼ of the time, half of the time, roughly ¾ of the time, almost all the time
Emotional demands	Does it happen that through work you come into close contact with the seriously ill or people with serious problems?	Not at all/rarely the last 3 months, a few days per month, one day per week, a few days per week, every day
Confrontation	Are you involved in any form of conflict or quarrel in your workplace with other people (eg, patients, customers, clients, passengers, students)?	Not at all the last 12 months, sometime the last 12 months, a few times the last 3 months, a few days per month, one day per week, a few days per week, every day

We imputed exposures in occupations that had fewer than 10 respondents in the surveys using exposures in occupations within a similar occupational coding group that had 10 or more respondents. A similar approach has previously been applied for constructing JEMs for job control, job demands and social support at work using the Swedish Work Environment Surveys.^23^

We linked the JEM to the study population using their occupational codes in 2005. We categorised each dimension into tertiles based on the distribution in the study population. Spearman correlations between the three dimensions were moderate, ranging between 0.46 and 0.51 (online supplemental table S1).

We listed the 20 occupations with the highest level of exposure to each of the three dimensions in men and women in online supplemental tables S2–S4. In both men and women, these occupations encompassed sectors of healthcare, education, service industry, hospitality, social work, legal professional, guards and transportation.

### Outcome

We extracted the first diagnosis of type 2 diabetes (ICD-10 code E11) from the national inpatient and outpatient registers and the cause of death register using both underlying and contributing causes of death, as well as the first prescription of antidiabetic drugs (ATC code A10) between 2006 and 2020.

### Covariates

Information on age, birth country, education and civil status was obtained from the LISA register. Birth country was categorised according to whether the individual was born in Sweden or not. Highest attained education was categorised as compulsory school only (≤9 years), vocational (10–11 years), upper secondary (12 years), post-secondary (13–14 years) and university (≥15 years). Civil status was categorised as married/partnered, unmarried, divorced and widowed.

To capture individuals’ early-life socioeconomic position (SEP), they were linked to their parents. We used information from the population and housing censuses from 1960 (for those born between 1941 and 1954), 1970 (for those born between 1955 and 1964), 1980 (for those born between 1965 and 1974) and 1990 (for those born between 1975 and 1989). Early-life SEP was estimated according to the father’s occupation, or mother’s occupation if the father’s was missing, and categorised as non-manual employees at a higher level, non-manual employees at an intermediate level, assistant non-manual employees, skilled manual workers, non-skilled manual workers, farmers, and those with no parental occupation documented.

Job control and social support at work in 2005 were assessed using JEMs based on the Swedish Work Environment Surveys (1997–2013). The questions in the surveys are shown in online supplemental table S5. We dichotomised them using the medians in the study population, respectively.^23^

### Statistical analysis

We explored baseline characteristics of the study population according to the outcome by the end of the follow-up period, as well as the levels of dimensions of person-related work.

We estimated the incidence rate of type 2 diabetes for each level of the three person-related work variables. Cox proportional hazard regression models with age as the underlying timescale were built to estimate hazard ratios (HRs) and 95% confidence intervals (CIs) for associations of the person-related work variables with the risk of type 2 diabetes. Person-time was counted from 1 January 2006 until the incidence of type 2 diabetes, death, emigration or end of the follow-up period on 31 December 2020, whichever came first.

We tested the effect modification of social support at work in the association between person-related work and type 2 diabetes by entering an interaction term of the person-related work variable and social support in the model. For this analysis, we omitted the medium level of the person-related work variable for simplification of interpretation. We used a likelihood ratio test (LRT) to test the overall significance of the interaction. Interaction that had p<0.05 was considered as moderating the association between the person-related work variable and type 2 diabetes.^24^ To further interpret the interaction, we estimated associations of combinations of person-related work variables and social support with type 2 diabetes.

It is known that men and women tend to hold different occupations and positions, and may have different types of exposures even within the same occupations. It is especially important for person-related work because many of the occupations are female dominated. Furthermore, it has been shown that the impact of work stress on type 2 diabetes appeared to be stronger in women than in men.^3 16^ Therefore, all analyses were done for men and women separately.

Model 1 was adjusted for age, birth year, civil status, birth country and early-life SEP. Model 2 was additionally adjusted for education. Model 3 was additionally adjusted for job control, because low job control has been found to be present in some person-related work and has been associated with type 2 diabetes as well.^3 4 10^

We explored the association in three age groups, 30–39, 40–49 and 50–60 years at baseline. Insulin prescriptions are becoming more common in patients with type 2 diabetes,^25^ but to avoid including participants with type 1 diabetes, an additional analysis was conducted in which insulin prescriptions (ATC code A10A) as a sole indication of type 2 diabetes were omitted (n=8000); that is, type 2 diabetes diagnosis (ICD-10 code E11) and non-insulin antidiabetic drugs (ATC code A10B) were taken into account for the outcome.

Data management and statistical analyses were done using STATA 17 (StataCorp LLC, College Station, TX, USA).

## Results

From 2006 to 2020 (over 41 767 442 person-years), 216 640 individuals (60.0% men) developed type 2 diabetes, of which 83.6% were identified through the drug register. In both men and women, those who developed type 2 diabetes, compared with those who did not, were older and more likely to be born outside Sweden and to have lower education and low job control. Their parents were less likely to be non-manual workers (table 2).

**Table 2:** Baseline characteristics according to incidence of type 2 diabetes by sex

Characteristics	Men (n=1 448 591)		Women (n=1 501 595)	
	With type 2 diabetes (n=130 183)	Without type 2 diabetes (n=1 318 408)	With type 2 diabetes (n=86 457)	Without type 2 diabetes (n=1 415 138)
Age				
30–39	13.9	36.3	15.9	33.6
40–49	31.3	32.7	30.0	32.9
50–60	54.8	31.0	54.1	33.5
Foreign born	16.4	11.1	18.4	12.3
Education years				
≤9	24.0	14.9	17.8	10.1
10–11	36.9	33.8	40.7	31.9
12	14.6	15.8	13.2	16.1
13–14	11.9	15.1	13.2	17.0
≥15	12.6	20.4	15.1	24.9
Civil status				
Married/partnered	52.3	49.7	55.1	53.3
Unmarried	31.1	39.1	23.5	30.6
Divorced	15.8	10.8	18.8	14.7
Widowed	0.8	0.4	2.6	1.4
Parents’ occupation				
Non-manual higher level	3.8	6.4	3.3	6.1
Non-manual intermediate level	13.2	18.3	12.1	17.5
Non-manual assistant	9.2	10.8	8.3	10.6
Skilled manual	23.8	23.4	24.2	23.3
Non-skilled manual	25.9	22.9	26.3	23.0
Farmer	5.6	5.4	5.5	5.7
No record	18.5	12.8	20.3	13.8
Low job control	59.8	50.8	64.5	53.5
Low social support at work	51.0	50.4	51.0	50.4

Data are given as percentages.

Women who had high exposures to general contact with people were younger, had higher education, less likely to be foreign born, and more likely to have low job control (online supplemental table S6).

In both men and women, those with high emotional demands were older, more likely to be foreign born and to have low job control and low social support at work (online supplemental table S7).

In both men and women, those who had high confrontation were older and had higher education. Women with high confrontation were more likely to have low social support (online supplemental table S8).

Table 3 shows the number of cases, follow-up time, incidence rates and HRs for type 2 diabetes by dimensions of person-related work in men and women separately. Across dimensions of person-related work, incidence rates for type 2 diabetes in men (5.61–7.19 per 1000 person-years) were higher than those in women (3.64–4.59 per 1000 person-years). In men, high exposures to the three dimensions, compared with low exposures, were associated with a 3–17% increased risk of type 2 diabetes in model 1. The effect estimates became stronger after controlling for education in model 2 (9–23% increased risk of type 2 diabetes). Controlling for job control in model 3 resulted in some alterations of effect estimates (14–20% increased risk of type 2 diabetes). In women, high exposures to the three dimensions were associated with a 7–28% increased risk of type 2 diabetes in model 2. However, the risk of type 2 diabetes in relation to general contact with people was no longer increased after controlling for job control in model 3 (HR 0.99, 95% CI 0.98 to 1.01), while effect estimates regarding the other two dimensions remained similar (HR 1.24 for emotional demands and HR 1.20 for confrontation).

**Table 3:** Number of cases, follow-up time, incidence rates (95% CI), and hazard ratios (95% CI) for type 2 diabetes by dimensions of person-related work

	No of cases	Follow-up (person-years)	Incidence rate per 1000 person-years (95% CI)	Model 1 HR (95% CI)	Model 2 HR (95% CI)	Model 3 HR (95% CI)
**Men**						
General contact with people						
Low	44 879	6 899 525	6.51 (6.45 to 6.57)	Ref	Ref	Ref
Medium	39 619	6 688 993.1	5.92 (5.87 to 5.98)	0.90 (0.89 to 0.92)	0.94 (0.92 to 0.95)	1.02 (1.01 to 1.04)
High	45 685	6 674 339.4	6.85 (6.78 to 6.91)	1.03 (1.02 to 1.05)	1.09 (1.08 to 1.11)	1.14 (1.12 to 1.15)
Emotional demands						
Low	38 360	6 842 587.4	5.61 (5.55 to 5.66)	Ref	Ref	Ref
Medium	44 406	6 825 111.9	6.51 (6.45 to 6.57)	1.12 (1.11 to 1.14)	1.11 (1.10 to 1.13)	1.06 (1.04 to 1.07)
High	47 417	6 595 158.2	7.19 (7.13 to 7.26)	1.17 (1.15 to 1.18)	1.23 (1.21 to 1.25)	1.20 (1.17 to 1.21)
Confrontation						
Low	42 628	6 796 552	6.27 (6.21 to 6.33)	Ref	Ref	Ref
Medium	42 788	6 735 418.3	6.35 (6.29 to 6.41)	1.00 (0.99 to 1.02)	1.02 (1.00 to 1.03)	1.06 (1.04 to 1.07)
High	44 767	6 730 887.3	6.65 (6.59 to 6.71)	1.03 (1.02 to 1.04)	1.12 (1.11 to 1.14)	1.15 (1.13 to 1.16)
**Women**						
General contact with people						
Low	28 937	7 238 061.1	4.00 (3.95 to 4.04)	Ref	Ref	Ref
Medium	30 921	7 213 167.3	4.29 (4.24 to 4.34)	1.07 (1.05 to 1.09)	1.11 (1.09 to 1.13)	1.13 (1.11 to 1.15)
High	26 599	7 053 356.1	3.77 (3.73 to 3.82)	0.97 (0.96 to 0.99)	1.07 (1.05 to 1.09)	0.99 (0.98 to 1.01)
Emotional demands						
Low	27 116	7 443 370.2	3.64 (3.60 to 3.69)	Ref	Ref	Ref
Medium	26 669	6 947 558.8	3.84 (3.79 to 3.89)	1.00 (0.98 to 1.02)	1.09 (1.08 to 1.11)	1.09 (1.07 to 1.11)
High	32 672	7 113 655.5	4.59 (4.54 to 4.64)	1.17 (1.15 to 1.18)	1.28 (1.26 to 1.30)	1.24 (1.22 to 1.26)
Confrontation						
Low	27 927	7 472 814.7	3.74 (3.69 to 3.78)	Ref	Ref	Ref
Medium	29 204	7 128 355.8	4.10 (4.05 to 4.14)	1.06 (1.04 to 1.08)	1.07 (1.05 to 1.09)	1.00 (0.98 to 1.02)
High	29 326	6 903 414.1	4.25 (4.20 to 4.30)	1.10 (1.09 to 1.12)	1.17 (1.16 to 1.19)	1.20 (1.18 to 1.22)

Model 1 adjusting for age, birth year, civil status, birth country and early-life socioeconomic position.

Model 2 adjusting for age, birth year, civil status, birth country, early-life socioeconomic position and education.

Model 3 adjusting for age, birth year, civil status, birth country, early-life socioeconomic position, education and job control.

In both men and women, there was statistically significant effect modification by social support for the associations of emotional demands and confrontation with type 2 diabetes (p<0.001 for the four LRTs). The associations between the two dimensions and type 2 diabetes were higher among those with low social support than those with high social support. The highest risk of type 2 diabetes was present in women with high emotional demands and low social support (HR 1.47, 95% CI 1.42 to 1.51) (table 4).

**Table 4:** Incidence rates (95% CI) and hazard ratios (95% CI) for type 2 diabetes by combinations of dimensions of person-related work and social support at work

		Incidence rate per 1000 person-years (95% CI)	Model 3 HR (95% CI)	P value of likelihood-ratio test
**Men**				
General contact with people	Social support at work			0.52
Low	High	6.54 (6.47 to 6.62)	Ref	
Low	Low	6.43 (6.33 to 6.54)	0.99 (0.97 to 1.01)	
High	High	6.38 (6.29 to 6.47)	1.09 (1.07 to 1.11)	
High	Low	7.24 (7.15 to 7.33)	1.10 (1.08 to 1.12)	
Emotional demands	Social support at work			<0.001
Low	High	5.42 (5.35 to 5.50)	Ref	
Low	Low	5.78 (5.70 to 5.86)	0.94 (0.92 to 0.96)	
High	High	7.07 (6.98 to 7.16)	1.16 (1.14 to 1.18)	
High	Low	7.30 (7.21 to 7.39)	1.23 (1.21 to 1.25)	
Confrontation	Social support at work			<0.001
Low	High	6.44 (6.36 to 6.52)	Ref	
Low	Low	6.02 (5.92 to 6.11)	0.90 (0.89 to 0.92)	
High	High	6.89 (6.80 to 6.98)	1.12 (1.10 to 1.14)	
High	Low	6.44 (6.36 to 6.53)	1.17 (1.14 to 1.19)	
**Women**				
General contact with people	Social support at work			0.47
Low	High	4.06 (4.00 to 4.12)	Ref	
Low	Low	3.90 (3.83 to 3.97)	1.01 (0.99 to 1.04)	
High	High	4.40 (4.33 to 4.47)	1.02 (0.99 to 1.04)	
High	Low	3.17 (3.12 to 3.23)	1.02 (0.99 to 1.05)	
Emotional demands	Social support at work			<0.001
Low	High	4.12 (4.07 to 4.18)	Ref	
Low	Low	2.60 (2.54 to 2.67)	0.88 (0.85 to 0.91)	
High	High	4.69 (4.61 to 4.78)	1.10 (1.07 to 1.13)	
High	Low	4.54 (4.48 to 4.60)	1.47 (1.42 to 1.51)	
Confrontation	Social support at work			<0.001
Low	High	3.83 (3.76 to 3.89)	Ref	
Low	Low	3.65 (3.59 to 3.71)	1.07 (1.05 to 1.10)	
High	High	4.42 (4.31 to 4.53)	1.04 (1.01 to 1.08)	
High	Low	4.21 (4.15 to 4.26)	1.18 (1.16 to 1.21)	

Model 3 adjusting for age, birth year, civil status, birth country, early-life socioeconomic position, education and job control.

The medium level of dimensions of person-related work was omitted in this analysis.

The observed associations were present in all three age groups (online supplemental tables S9 and S10). Our results remained robust in the sensitivity analysis where insulin prescriptions (ATC code A10A) as a sole indication of type 2 diabetes were omitted (online supplemental tables S11 and S12).

## Discussion

Using a nationwide register-based cohort, we investigated the risk of type 2 diabetes in relation to three dimensions of person-related work. We found that in both women and men, high levels of general contact with people, emotional demands and confrontation were respectively associated with an increased risk of type 2 diabetes. However, in women, the risk related to general contact with people was no longer increased after taking job control into account. Furthermore, social support at work appeared to modify the associations: the risk of type 2 diabetes in relation to emotional demands and confrontation was higher among those who received low social support at work than those who received high social support.

To our knowledge, the current study is the first that focused on person-related work and the risk of type 2 diabetes. Previous studies have linked person-related work and emotional demands at work to a higher risk of mental health problems^22 26 27^ and sickness absence.^28 29^ Our analyses based on a similar study sample and methodology showed that high exposures to the three dimensions were associated with a higher risk of cardiovascular disease.^30^

Our findings that high exposures to the three dimensions of person-related work were associated with a higher risk of type 2 diabetes were generally in line with our hypotheses. They support the notion that stress can occur when working with people and affect workers’ metabolic health.^9^

With regards to having contact with people at work, there are expectations for emotional management where workers are required to express or hide emotions according to societal, occupational and organisational norms.^15^ It is especially stressful when the displayed emotion and the genuinely felt emotion are not aligned.^8^ Nevertheless, arguably some degree of effort may be necessary for regulating emotions even if there is congruence between the worker’s felt emotion and the organisationally required emotion. One example would be that, instead of floods of tears, workers are supposed to express their sympathy to clients with appropriate facial display and tone of voice.^15^

Occupations that involve contact with individuals who are ill or in problematic situations may be especially emotionally demanding. Workers in human service occupations, such as healthcare professionals and social workers, take responsibility for the fundamental human needs of clients and witness human suffering, and in most cases, there is no reciprocity in relations with clients and patients.^31^ These are potential stressors that can result in compassion fatigue, burnout^32^ and mental health problems among workers in such occupations.^22^

Furthermore, it has been shown that work-related threats and violence are present in person-related work, especially in the social and healthcare sectors.^33 34^ Our findings provided evidence that confrontation, including conflict and quarrel with people, can contribute to the risk of type 2 diabetes. Similarly, a previous study also found the association between workplace violence and bullying and a higher type 2 diabetes risk, although the source of violence and bullying was not specified in the study.^6^

We observed that the association between general contact with people and type 2 diabetes was attenuated after taking job control into account in women, but not in men. Previous studies showed that low work-time control^10^ and low decision authority^35^ were involved in person-related work. A meta-analysis based on published studies showed that low job control was associated with type 2 diabetes in women, but not in men,^3^ while another meta-analysis based on individual participant data observed the association in both men and women.^4^ In our data, while we observed that low job control was associated with a higher risk of type 2 diabetes in both sexes, women who had high exposure to general contact with people were more likely to have low job control, but this was not the case in men (online supplemental table S6).

In line with our hypotheses, we identified the effect modification of social support at work in the association between person-related work and type 2 diabetes, where the effect estimates were higher among those who received low social support at work. The highest effect estimate was observed in women with high emotional demands and low social support at work. These findings echo what was shown in a previous study regarding the interaction between work stress and emotional support at work in relation to type 2 diabetes in women.^16^ On the other hand, a heightened risk of type 2 diabetes related to emotional demands and confrontation was still present among workers with high social support. Of note, items used to assess social support at work in the Swedish Work Environment Surveys were generic and not specific to scenarios and demands in person-related work. Therefore, more research into developing support systems that are specific to different contexts in person-related work is needed.

The biological mechanisms underlying the association between person-related work and type 2 diabetes may involve biological responses to repeated and chronic stress that affects the neuroendocrine system by activation of the central sympathetic nervous system and hypothalamus–pituitary–adrenal axis, leading to excessive cortisol production, increased insulin resistance, and decreased insulin secretion and sensitivity.^9^ In addition, chronic stress can increase pro-inflammatory cytokines, which impair insulin signalling and functioning.^9 36^ With insufficient social support at work, stress in person-related work may worsen and exert a greater impact on these biological alterations.

Strengths of the study include the use of a nationwide, representative sample which can help reduce selection or attrition bias, and the use of JEMs to assess work-related exposures providing measures less affected by other factors and minimising reporting bias. Specifically, emotional demands assessed in the study represented content-related emotional demands, which, compared with perceived emotional demands, are less likely to be influenced by mental health issues.^29^ Moreover, instead of focusing on specific occupations, focusing on dimensions allowed us to categorise occupations based on common characteristics regarding exposures in person-related work. Furthermore, type 2 diabetes was identified based on both diagnosis in patient and death registers and prescriptions in the drug register, which enhanced the coverage of cases.

This study also has limitations. Despite the strength mentioned above, JEMs assess work-related exposures on the occupational level and are unable to capture variations in individuals’ experiences or feelings or work environment within a given occupation. These are likely to result in an underestimation of the true associations due to non-differential misclassification of exposures at work. Also, we only used one item for each dimension of person-related work and thus may have missed other aspects related to the dimensions. Additionally, we did not have information on individuals’ whole occupational history, and thus the duration of exposure was uncertain. We also did not consider the possible change of occupations; however, a previous study using the same cohort showed that psychosocial work environment assessed at the occupational level is quite stable over time.^37^

Moreover, workers in person-related jobs may be extroverted and sociable. It has been shown that these personality traits are associated with higher alcohol consumption,^38^ which may in turn increase the workers’ risk of developing type 2 diabetes.^39^ Therefore, we were unable to rule out the potential confounding related to workers’ personality. On the other hand, unhealthy behaviours may also be a strategy for workers to cope with stress in person-related work.^15 16^ We were unable to disentangle biological (ie, pathological changes due to the stress response) and behavioural mechanisms underlying the observed associations due to the lack of information on lifestyle factors.

In conclusion, person-related work is associated with an increased risk of type 2 diabetes, and lacking social support at work may further amplify the association. Our findings highlight the impact of demands and challenges in person-related work on workers’ metabolic health. Future studies are warranted to explore mechanisms (eg, biological or behavioural mechanisms) underlying the associations and develop preventive strategies aiming to lower health hazards in person-related work.

## Supplementary material

10.1136/oemed-2025-110088 online supplemental file 1

## Data Availability

Data may be obtained from a third party and are not publicly available.
