# Supplementary material for: Person-related work and the risk of type 2 diabetes: a Swedish register-based cohort study
Source: Occup Environ Med. 2025 Jun 24;82(4):e110088. doi: 10.1136/oemed-2025-110088 (PMC12322397; doi:10.1136/oemed-2025-110088)
Supplement: online supplemental file 1 [file oemed-82-4-s001.docx]

Supplementary Material

Table S1. Spearman correlations between the three dimensions of person-related work

|  | General contact with people | Emotional demands | Confrontation |
| --- | --- | --- | --- |
| General contact with people | 1 |  |  |
| Emotional demands | 0.51 | 1 |  |
| Confrontation | 0.46 | 0.50 | 1 |

Table S2. Twenty occupations with the highest exposure to general contact with people by sex

| **Men** | |
| --- | --- |
| Occupational code | Occupational title |
| 3234 | Psychiatric nurses |
| 2235 | District nurses |
| 2234 | Paediatric nurses |
| 3225 | Dental hygienists |
| 2222 | Dentists |
| 3239 | Nursing associate professionals not elsewhere classified |
| 2233 | Emergency room nurses |
| 2231 | Midwives |
| 3226 | Physiotherapists and related associate professionals |
| 3221 | Occupational therapists |
| 3232 | Surgery nurses |
| 3233 | Geriatric nurses |
| 3231 | Nursing associate professionals |
| 3223 | Dieticians |
| 3224 | Optometrists and opticians |
| 3228 | Pharmaceutical assistants |
| 3229 | Other therapists |
| 4221 | Travel agency and related clerks |
| 5111 | Travel attendants and travel stewards |
| 5226 | Car, boat and caravan salesmen |
| **Women** | |
| 5112 | Transport conductors |
| 2222 | Dentists |
| 8322 | Bus and tram drivers |
| 2233 | Emergency room nurses |
| 3225 | Dental hygienists |
| 3224 | Optometrists and opticians |
| 5111 | Travel attendants and travel stewards |
| 3417 | Appraisers, valuers and auctioneers |
| 2235 | District nurses |
| 2231 | Midwives |
| 5135 | Dental nurses |
| 3229 | Other therapists |
| 3228 | Pharmaceutical assistants |
| 5225 | Gas station managers |
| 2225 | Speech therapists |
| 2229 | Health care professionals not elsewhere classified |
| 2460 | Religious professionals |
| 8311 | Train drivers |
| 5142 | Undertakers |
| 8321 | Car and taxi drivers |

Table S3. Twenty occupations with the highest exposure to emotional demands by sex

| **Men** | |
| --- | --- |
| Occupational code | Occupational title |
| 3233 | Geriatric nurses |
| 3232 | Surgery nurses |
| 3231 | Nursing associate professionals |
| 2233 | Emergency room nurses |
| 2231 | Midwives |
| 5153 | Prison guards |
| 5132 | Assistant nurses and hospital ward assistants |
| 3234 | Psychiatric nurses |
| 2221 | Medical doctors |
| 2235 | District nurses |
| 2234 | Paediatric nurses |
| 3461 | Social workers and related associate professionals |
| 5134 | Carers and caregivers |
| 3239 | Nursing associate professionals not elsewhere classified |
| 5133 | Nursing assistants, personal assistants |
| 2225 | Speech therapists |
| 2229 | Health care professionals not elsewhere classified |
| 2491 | Psychologists |
| 1318 | Managers of small enterprises in healthcare and social work |
| 4214 | Pawnbrokers and moneylenders |
| **Women** | |
| 5153 | Prison guards |
| 3234 | Psychiatric nurses |
| 2233 | Emergency room nurses |
| 3233 | Geriatric nurses |
| 3231 | Nursing associate professionals |
| 3235 | Radiology nurses |
| 3239 | Nursing associate professionals not elsewhere classified |
| 3232 | Surgery nurses |
| 2235 | District nurses |
| 5132 | Assistant nurses and hospital ward assistants |
| 3221 | Occupational therapists |
| 2221 | Medical doctors |
| 2492 | Social workers and curators |
| 2491 | Psychologists |
| 3228 | Pharmaceutical assistants |
| 2225 | Speech therapists |
| 2229 | Health care professionals not elsewhere classified |
| 3226 | Physiotherapists and related associate professionals |
| 5133 | Nursing assistants, personal assistants |
| 3461 | Social workers and related associate professionals |

Table S4. Twenty occupations with the highest exposure to confrontation by sex

| **Men** | |
| --- | --- |
| Occupational code | Occupational title |
| 5153 | Prison guards |
| 3310 | Pre-primary education teaching associate professionals |
| 3461 | Social workers and related associate professionals |
| 4224 | Transport information clerks |
| 2340 | Special education teaching professionals |
| 5131 | Child-care workers |
| 5111 | Travel attendants and travel stewards |
| 3450 | Police officers and detectives |
| 2330 | Primary education teaching professionals |
| 1318 | Managers of small enterprises in healthcare and social work |
| 5112 | Transport conductors |
| 5134 | Carers and caregivers |
| 3234 | Psychiatric nurses |
| 4214 | Pawnbrokers and moneylenders |
| 4215 | Debt-collectors and related workers |
| 4213 | Croupiers |
| 2323 | Teacher in aesthetic and practical subjects |
| 8340 | Ships' deck crews and related workers |
| 5224 | Kiosk managers |
| 3472 | Radio, television and other announcers |
| **Women** | |
| 5153 | Prison guards |
| 4215 | Debt-collectors and related workers |
| 4214 | Pawnbrokers and moneylenders |
| 2330 | Primary education teaching professionals |
| 2340 | Special education teaching professionals |
| 3234 | Psychiatric nurses |
| 1316 | Managers of small enterprises in public administration |
| 3450 | Police officers and detectives |
| 3310 | Pre-primary education teaching associate professionals |
| 3461 | Social workers and related associate professionals |
| 5112 | Transport conductors |
| 5149 | Other personal services workers not elsewhere classified |
| 9121 | Maids |
| 5134 | Carers and caregivers |
| 3417 | Appraisers, valuers and auctioneers |
| 3152 | Safety, health and quality inspectors |
| 5151 | Firefighters |
| 5152 | Watchmen and security guards |
| 3441 | Customs and border inspectors |
| 2323 | Teacher in aesthetic and practical subjects |

Table S5. Translated items used for job control and social support at work in the Job Exposure Matrices

| Job control | Can you partially decide when tasks should be done? |
| --- | --- |
|  | Do you have the opportunity to decide your own work pace? |
|  | Can you take short breaks to talk pretty much any time? |
|  | Are you ever involved in deciding how your work is organized? |
|  | Is there any apprenticeship or introductory training required at the workplace (besides education or course) before you can do your job? |
|  | Does the work require you to repeat the same work steps many times an hour? |
|  | Do you spend quite some time during the day trying to understand or solving difficult problems? |
|  | Does the work offer you the possibility to learn something new and to develop in this occupation? |
| Social support at work | Do you have the opportunity to get support and encouragement from co-workers, when the work feels difficult? |
|  | Do you have the opportunity to get support and encouragement from managers, when the work feels difficult? |
|  | If the tasks feel so difficult, do you then have the opportunity to get advice or help? |
|  | Does it happen that your boss shows appreciation for something you have done? |
|  | Do other people show appreciation for something you have done? |

Table S6. Baseline characteristics according to levels of general contact with people by sex

|  | Men | | | Women | | |
| --- | --- | --- | --- | --- | --- | --- |
| General contact with people | Low  (N=497,814) | Medium  (N=481,705) | High  (N=483,842) | Low  (N=509,229) | Medium  (N=508,177) | High  (N=493,304) |
| Characteristics | % | % | % | % | % | % |
| Age |  |  |  |  |  |  |
| 30-39 | 36.1 | 33.2 | 33.0 | 32.4 | 31.4 | 33.8 |
| 40-49 | 33.1 | 32.2 | 32.2 | 32.7 | 33.1 | 32.2 |
| 50-60 | 30.8 | 34.6 | 34.8 | 34.9 | 35.5 | 34.0 |
| Foreign born | 13.5 | 9.2 | 12.1 | 13.7 | 12.7 | 11.6 |
| Education years |  |  |  |  |  |  |
| ≤9 | 19.3 | 14.2 | 13.9 | 14.9 | 10.3 | 6.4 |
| 10-11 | 37.6 | 35.0 | 29.8 | 31.8 | 34.6 | 30.7 |
| 12 | 14.7 | 16.3 | 16.1 | 19.2 | 14.3 | 14.4 |
| 13-14 | 13.3 | 14.0 | 16.9 | 14.7 | 21.3 | 14.4 |
| ≥15 | 15.1 | 25.5 | 23.3 | 19.4 | 19.5 | 34.1 |
| Civil status |  |  |  |  |  |  |
| Married/Partnered | 47.3 | 51.4 | 51.3 | 53.0 | 53.5 | 53.8 |
| Unmarried | 41.9 | 37.2 | 35.6 | 31.0 | 29.6 | 29.9 |
| Divorced | 10.4 | 10.9 | 12.6 | 14.5 | 15.4 | 15.0 |
| Widowed | 0.4 | 0.5 | 0.5 | 1.5 | 1.5 | 1.3 |
| Parents’ occupation |  |  |  |  |  |  |
| Non-manual higher level | 4.5 | 6.7 | 7.4 | 6.3 | 5.3 | 6.1 |
| Non-manual intermediate level | 15.8 | 19.2 | 18.3 | 18.1 | 16.2 | 17.2 |
| Non-manual assistant | 8.6 | 10.8 | 12.7 | 10.4 | 10.4 | 10.7 |
| Skilled manual | 24.3 | 25.0 | 21.1 | 22.8 | 23.8 | 23.6 |
| Non-skilled manual | 24.7 | 22.3 | 22.6 | 22.2 | 23.9 | 23.3 |
| Farmer | 7.1 | 5.0 | 4.1 | 5.2 | 5.8 | 6.0 |
| No record | 15.0 | 11.0 | 13.8 | 15.0 | 14.6 | 13.1 |
| Low job control | 59.8 | 37.6 | 57.5 | 50.3 | 45.5 | 67.1 |
| Low social support | 33.3 | 64.4 | 54.2 | 39.2 | 66.3 | 51.1 |

Table S7. Baseline characteristics according to levels of emotional demands by sex

|  | Men |  |  | Women |  |  |
| --- | --- | --- | --- | --- | --- | --- |
| Emotional demands | Low  (N=489,506) | Medium  (N=493,269) | High  (N=480,586) | Low  (N=522,211) | Medium  (N=486,591) | High  (N=501,908) |
| Characteristics | % | % | % | % | % | % |
| Age |  |  |  |  |  |  |
| 30-39 | 36.2 | 34.2 | 31.8 | 36.7 | 31.6 | 29.1 |
| 40-49 | 32.9 | 32.9 | 31.8 | 31.9 | 32.3 | 33.9 |
| 50-60 | 30.9 | 32.9 | 36.4 | 31.4 | 36.1 | 37.0 |
| Foreign born | 8.9 | 9.9 | 16.1 | 12.3 | 12.6 | 13.2 |
| Education years |  |  |  |  |  |  |
| ≤9 | 15.7 | 18.1 | 13.7 | 15.0 | 10.4 | 6.3 |
| 10-11 | 36.7 | 36.8 | 28.8 | 33.5 | 27.2 | 36.3 |
| 12 | 15.2 | 16.6 | 15.3 | 20.6 | 13.8 | 13.1 |
| 13-14 | 14.8 | 13.0 | 16.5 | 12.9 | 20.6 | 17.3 |
| ≥15 | 17.6 | 15.5 | 25.7 | 18.0 | 28.0 | 27.0 |
| Civil status |  |  |  |  |  |  |
| Married/Partnered | 49.7 | 49.6 | 50.7 | 51.8 | 55.5 | 53.2 |
| Unmarried | 40.1 | 38.9 | 35.8 | 33.1 | 28.8 | 28.5 |
| Divorced | 9.8 | 11.0 | 13.0 | 13.7 | 14.3 | 16.8 |
| Widowed | 0.4 | 0.5 | 0.5 | 1.4 | 1.4 | 1.5 |
| Parents’ occupation |  |  |  |  |  |  |
| Non-manual higher level | 5.8 | 5.6 | 7.1 | 6.4 | 6.2 | 5.2 |
| Non-manual intermediate level | 18.5 | 17.3 | 17.5 | 18.2 | 18.1 | 15.1 |
| Non-manual assistant | 9.8 | 11.2 | 11.1 | 10.9 | 11.0 | 9.5 |
| Skilled manual | 25.2 | 24.1 | 21.0 | 23.2 | 22.9 | 24.1 |
| Non-skilled manual | 23.1 | 25.0 | 21.5 | 22.6 | 22.3 | 24.6 |
| Farmer | 7.0 | 5.2 | 4.1 | 5.2 | 5.4 | 6.3 |
| No record | 10.6 | 11.6 | 17.7 | 13.5 | 14.1 | 15.2 |
| Low job control | 41.7 | 47.5 | 66.2 | 51.7 | 44.2 | 66.4 |
| Low social support | 51.6 | 47.1 | 52.8 | 31.4 | 61.7 | 64.6 |

Table S8. Baseline characteristics according to levels of confrontation by sex

|  | Men |  |  | Women |  |  |
| --- | --- | --- | --- | --- | --- | --- |
| Confrontation | Low  (N=488,841) | Medium  (N=497,049) | High  (N=477,471) | Low  (N=524,661) | Medium  (N=500,675) | High  (N=485,374) |
| Characteristics | % | % | % | % | % | % |
| Age |  |  |  |  |  |  |
| 30-39 | 35.7 | 33.5 | 33.1 | 34.7 | 31.2 | 31.5 |
| 40-49 | 32.9 | 33.1 | 31.6 | 32.5 | 33.0 | 32.5 |
| 50-60 | 31.4 | 33.4 | 35.3 | 32.8 | 35.8 | 36.0 |
| Foreign born | 11.1 | 9.9 | 13.9 | 14.4 | 11.6 | 11.9 |
| Education years |  |  |  |  |  |  |
| ≤9 | 18.1 | 17.5 | 11.7 | 14.6 | 8.8 | 8.1 |
| 10-11 | 40.1 | 35.0 | 27.2 | 27.9 | 38.2 | 31.3 |
| 12 | 15.0 | 15.8 | 16.3 | 17.7 | 18.4 | 11.4 |
| 13-14 | 12.5 | 12.9 | 19.0 | 14.9 | 14.4 | 21.5 |
| ≥15 | 14.3 | 18.8 | 25.8 | 24.9 | 20.2 | 27.7 |
| Civil status |  |  |  |  |  |  |
| Married/Partnered | 47.4 | 50.9 | 51.6 | 53.2 | 53.5 | 53.6 |
| Unmarried | 41.6 | 37.5 | 35.6 | 31.2 | 29.9 | 29.3 |
| Divorced | 10.5 | 11.1 | 12.3 | 14.2 | 15.1 | 15.6 |
| Widowed | 0.5 | 0.5 | 0.5 | 1.4 | 1.5 | 1.5 |
| Parents’ occupation |  |  |  |  |  |  |
| Non-manual higher level | 4.9 | 6.0 | 7.7 | 7.5 | 5.1 | 5.1 |
| Non-manual intermediate level | 16.2 | 17.6 | 19.5 | 18.5 | 16.3 | 16.6 |
| Non-manual assistant | 9.2 | 10.7 | 12.2 | 10.4 | 10.5 | 10.5 |
| Skilled manual | 26.2 | 23.2 | 21.0 | 21.7 | 24.4 | 24.1 |
| Non-skilled manual | 25.0 | 24.1 | 20.4 | 21.0 | 24.5 | 24.1 |
| Farmer | 5.7 | 6.7 | 3.8 | 5.3 | 6.0 | 5.8 |
| No record | 12.8 | 11.7 | 15.4 | 15.6 | 13.2 | 13.8 |
| Low job control | 61.6 | 41.7 | 52.0 | 44.3 | 74.3 | 44.1 |
| Low social support | 39.1 | 57.5 | 54.8 | 49.2 | 29.7 | 78.7 |

Table S9. Hazard ratios (95% CI) for type 2 diabetes by dimensions of person-related work and age at baseline

|  | Age 30-39 | Age 40-49 | Age 50-60 |
| --- | --- | --- | --- |
| General contact with people | Model 3  HR (95% CI) | Model 3  HR (95% CI) | Model 3  HR (95% CI) |
| Low | Ref | Ref | Ref |
| Medium | **1.10 (1.07-1.13)** | 1.02 (1.00-1.04) | **1.04 (1.02-1.05)** |
| High | **1.09 (1.06-1.12)** | **1.08 (1.06-1.10)** | **1.08 (1.06-1.09)** |
| Emotional demands |  |  |  |
| Low | Ref | Ref | Ref |
| Medium | **1.14 (1.11-1.17)** | **1.11 (1.09-1.14)** | **1.07 (1.06-1.09)** |
| High | **1.41 (1.40-1.45)** | **1.24 (1.21-1.26)** | **1.17 (1.15-1.19)** |
| Confrontation |  |  |  |
| Low | Ref | Ref | Ref |
| Medium | **1.06 (1.03-1.09)** | 1.01 (0.99-1.03) | **1.02 (1.00-1.03)** |
| High | **1.28 (1.25-1.32)** | **1.18 (1.16-1.20)** | **1.14 (1.12-1.16)** |

Model 3 adjusting for age, birth year, civil status, birth country, early-life socioeconomic position, education, and job control

Table S10. Hazard ratios (95% CI) for type 2 diabetes by combinations of dimensions of person-related work and social support at work and age at baseline

|  |  | Age 30-39 | | Age 40-49 | | Age 50-60 | |
| --- | --- | --- | --- | --- | --- | --- | --- |
|  |  | Model 3  HR (95% CI) | p-value of likelihood-ratio test | Model 3  HR (95% CI) | p-value of likelihood-ratio test | Model 3  HR (95% CI) | p-value of likelihood-ratio test |
| General contact with people | Social support at work |  | <0.001 |  | <0.001 |  | <0.001 |
| Low | High | Ref |  | Ref |  | Ref |  |
| Low | Low | 0.96 (0.93-1.00) |  | **0.92 (0.90-0.95)** |  | 1.00 (0.98-1.02) |  |
| High | High | **1.04 (1.01-1.08)** |  | 1.00 (0.98-1.03) |  | **1.03 (1.01-1.05)** |  |
| High | Low | **1.19 (1.14-1.25)** |  | **1.21 (1.18-1.25)** |  | **1.14 (1.11-1.16)** |  |
| Emotional demands | Social support at work |  | <0.001 |  | <0.05 |  | <0.001 |
| Low | High | Ref |  | Ref |  | Ref |  |
| Low | Low | 1.00 (0.96-1.04) |  | **1.03 (1.00-1.06)** |  | **1.08 (1.06-1.11)** |  |
| High | High | **1.37 (1.33-1.42)** |  | **1.23 (1.20-1.26)** |  | **1.20 (1.18-1.23)** |  |
| High | Low | **1.48 (1.42-1.54)** |  | **1.22 (1.19-1.26)** |  | **1.11 (1.09-1.13)** |  |
| Confrontation | Social support at work |  | <0.05 |  | <0.001 |  | <0.001 |
| Low | High | Ref |  | Ref |  | Ref |  |
| Low | Low | **0.95 (0.92-0.99)** |  | **0.89 (0.87-0.91)** |  | **0.97 (0.95-0.99)** |  |
| High | High | **1.24 (1.19-1.28)** |  | **1.14 (1.11-1.18)** |  | **1.17 (1.14-1.20)** |  |
| High | Low | **1.30 (1.25-1.35)** |  | **1.31 (1.18-1.24)** |  | **1.10 (1.08-1.12)** |  |

Model 3 adjusting for age, birth year, civil status, birth country, early-life socioeconomic position, education, and job control

Note: the medium level of dimensions of person-related work was omitted in this analysis

Table S11. Number of cases, follow up time, incidence rates (95% CI), and hazard ratios (95% CI) for type 2 diabetes by dimensions of person-related work, excluding type 2 diabetes cases indicated solely by insulin prescriptions (ATC code A10A)

| **Men** | n cases | Follow up (person-years) | Incidence rate per 1,000 person-years (95% CI) | Model 1  HR (95% CI) | Model 2  HR (95% CI) | Model 3  HR (95% CI) |
| --- | --- | --- | --- | --- | --- | --- |
| General contact with people | | | | | | |
| Low | 43,354 | 6,885,743.8 | 6.30 (6.24-6.36) | Ref | Ref | Ref |
| Medium | 38,305 | 6,677,113 | 5.74 (5.68-5.80) | **0.90 (0.89-0.92)** | **0.94 (0.92-0.95)** | **1.02 (1.01-1.04)** |
| High | 44,220 | 6,661,533.9 | 6.64 (6.58-6.70) | **1.03 (1.02-1.05)** | **1.09 (1.08-1.11)** | **1.14 (1.12-1.16)** |
| Emotional demands | | | | | | |
| Low | 37,076 | 6,831,041.9 | 5.43 (5.37-5.48) | Ref | Ref | Ref |
| Medium | 42,960 | 6,812,202.6 | 6.31 (6.25-6.37) | **1.12 (1.11-1.14)** | **1.11 (1.10-1.13)** | **1.06 (1.04-1.07)** |
| High | 45,843 | 6,581,146.1 | 6.97 (6.90-7.03) | **1.17 (1.15-1.18)** | **1.23 (1.21-1.25)** | **1.19 (1.17-1.21)** |
| Confrontation | | | | | | |
| Low | 41,223 | 6,783,919.1 | 6.08 (6.02-6.14) | Ref | Ref | Ref |
| Medium | 41,370 | 6,722,653.2 | 6.15 (6.10-6.21) | 1.00 (0.99-1.02) | **1.02 (1.00-1.03)** | **1.06 (1.04-1.07)** |
| High | 43,286 | 6,717,818.3 | 6.44 (6.38-6.51) | **1.03 (1.02-1.04)** | **1.12 (1.11-1.14)** | **1.15 (1.13-1.16)** |
| **Women** | | | | | | |
| General contact with people | | | | | | |
| Low | 27,782 | 7,228,987.7 | 3.84 (3.80-3.89) | Ref | Ref | Ref |
| Medium | 29,655 | 7,203,448.5 | 4.12 (4.07-4.16) | **1.07 (1.05-1.09)** | **1.11 (1.09-1.13)** | **1.13 (1.11-1.15)** |
| High | 25,324 | 7,043,294.8 | 3.60 (3.55-3.64) | **0.97 (0.95-0.98)** | **1.07 (1.05-1.09)** | 0.99 (0.97-1.01) |
| Emotional demands | | | | | | |
| Low | 25,977 | 7,434,385.7 | 3.49 (3.45-3.54) | Ref | Ref | Ref |
| Medium | 25,519 | 6,938,586.5 | 3.68 (3.63-3.72) | 1.00 (0.98-1.02) | **1.09 (1.07-1.11)** | **1.09 (1.07-1.11)** |
| High | 31,265 | 7,102,758.9 | 4.40 (4.35-4.45) | **1.16 (1.14-1.18)** | **1.28 (1.26-1.30)** | **1.24 (1.22-1.26)** |
| Confrontation | | | | | | |
| Low | 26,675 | 7,462,912.4 | 3.57 (3.53-3.62) | Ref | Ref | Ref |
| Medium | 27,972 | 7,118,876.7 | 3.93 (3.88-3.98) | **1.06 (1.05-1.08)** | **1.07 (1.05-1.09)** | 1.00 (0.98-1.02) |
| High | 28,114 | 6,893,941.9 | 4.08 (4.03-4.13) | **1.11 (1.09-1.12)** | **1.18 (1.16-1.20)** | **1.20 (1.18-1.23)** |

Model 1 adjusting for age, birth year, civil status, birth country, and early-life socioeconomic position

Model 2 adjusting for age, birth year, civil status, birth country, early-life socioeconomic position, and education

Model 3 adjusting for age, birth year, civil status, birth country, early-life socioeconomic position, education, and job control

Table S12. Incidence rates (95% CI) and hazard ratios (95% CI) for type 2 diabetes by combinations of dimensions of person-related work and social support at work, excluding type 2 diabetes cases indicated solely by insulin prescriptions (ATC code A10A)

| **Men** |  | Incidence rate per 1,000 person-years (95% CI) | Model 3  HR (95% CI) | p-value of likelihood-ratio test |
| --- | --- | --- | --- | --- |
| General contact with people | Social support at work |  |  | 0.61 |
| Low | High | 6.33 (6.26-6.40) | Ref |  |
| Low | Low | 6.23 (6.13-6.33) | 0.99 (0.97-1.01) |  |
| High | High | 6.18 (6.09-6.27) | **1.09 (1.07-1.11)** |  |
| High | Low | 7.03 (6.94-7.12) | **1.10 (1.08-1.13)** |  |
| Emotional demands | Social support at work |  |  | <0.001 |
| Low | High | 5.24 (5.16-5.32) | Ref |  |
| Low | Low | 5.61 (5.53-5.69) | **0.94 (0.92-0.96)** |  |
| High | High | 6.85 (6.76-6.94) | **1.16 (1.14-1.19)** |  |
| High | Low | 7.07 (6.98-7.16) | **1.23 (1.20-1.25)** |  |
| Confrontation | Social support at work |  |  | <0.001 |
| Low | High | 6.24 (6.16-6.31) | Ref |  |
| Low | Low | 5.83 (5.74-5.92) | **0.90 (0.89-0.92)** |  |
| High | High | 6.68 (6.59-6.77) | **1.12 (1.10-1.14)** |  |
| High | Low | 6.24 (6.16-6.33) | **1.17 (1.15-1.20)** |  |
| **Women** |  | Incidence rate per 1,000 person-years (95% CI) | Model 3  HR (95% CI) | p-value of likelihood-ratio test |
| General contact with people | Social support at work |  |  | 0.55 |
| Low | High | 3.91 (3.85-3.97) | Ref |  |
| Low | Low | 3.74 (3.67-3.81) | 1.01 (0.99-1.04) |  |
| High | High | 4.22 (4.15-4.29) | 1.02 (0.99-1.04) |  |
| High | Low | 3.01 (2.95-3.06) | 1.01 (0.98-1.04) |  |
| Emotional demands | Social support at work |  |  | <0.001 |
| Low | High | 3.97 (3.91-4.02) | Ref |  |
| Low | Low | 2.47 (2.41-2.54) | **0.88 (0.85-0.91)** |  |
| High | High | 4.50 (4.42-4.59) | **1.09 (1.07-1.12)** |  |
| High | Low | 4.35 (4.29-4.41) | **1.47 (1.43-1.51)** |  |
| Confrontation | Social support at work |  |  | <0.001 |
| Low | High | 3.67 (3.61-3.73) | Ref |  |
| Low | Low | 3.48 (3.42-3.54) | **1.07 (1.05-1.10)** |  |
| High | High | 4.24 (4.13-4.34) | **1.04 (1.01-1.08)** |  |
| High | Low | 4.04 (3.98-4.09) | **1.19 (1.16-1.22)** |  |

Model 3 adjusting for age, birth year, civil status, birth country, early-life socioeconomic position, education, and job control

Note: the medium level of dimensions of person-related work was omitted in this analysis
